# Quality of life among symptomatic compared to PSA-detected prostate cancer survivors - results from a UK wide patient-reported outcomes study

**DOI:** 10.1186/s12885-019-6164-5

**Published:** 2019-10-15

**Authors:** David W. Donnelly, Linda C. Vis, Therese Kearney, Linda Sharp, Damien Bennett, Sarah Wilding, Amy Downing, Penny Wright, Eila Watson, Richard Wagland, William R. Cross, Malcolm D. Mason, Sabine Siesling, Jeannette G. van Manen, Adam W. Glaser, Anna Gavin

**Affiliations:** 1 Northern Ireland Cancer Registry, Centre for Public Health, Queen’s University Belfast, Mulhouse Building, Grosvenor Road, Belfast, BT12 6DP Northern Ireland, UK; 2 Department of Health Technology & Services Research, University of Twente, Enschede, The Netherlands; 3 Institute of Health & Society, Newcastle University, Newcastle-upon-Tyne, England; 4 Leeds Institute of Medical Research at St James’s, University of Leeds, Leeds, England; 5 Leeds Institute of Data Analytics, University of Leeds, Leeds, England; 6 Department of Midwifery, Community and Public Health, School of Nursing and Midwifery, Oxford Brookes University, Oxford, England; 7 Faculty of Health Sciences, University of Southampton, Southampton, England; 8 Department of Urology, St James’s University Hospital, Leeds, England; 9 Division of Cancer and Genetics, School of Medicine, Cardiff University, Cardiff, Wales; 10 Netherlands Comprehensive Cancer Organisation, Utrecht, The Netherlands

**Keywords:** Prostate cancer, PSA, Symptoms, Presentation, Patient-reported outcomes, Quality of life

## Abstract

**Background:**

Quality of life among prostate cancer survivors varies by socio-demographic factors and treatment type received; however, less in known about differences in functional outcomes by method of presentation. We investigate differences in reported urinary, bowel, sexual and hormone-related problems between symptomatic and PSA-detected prostate cancer survivors.

**Methods:**

A UK wide cross-sectional postal survey of prostate cancer survivors conducted 18-42 months post-diagnosis. Questions were included on presentation method and treatment. Functional outcome was determined using the EPIC-26 questionnaire. Reported outcomes were compared for symptomatic and PSA-detected survivors using ANOVA and multivariable log-linear regression.

**Results:**

Thirty-five thousand eight hundred twenty-three men responded (response rate: 60.8%). Of these, 31.3% reported presenting via PSA test and 59.7% symptomatically. In multivariable analysis, symptomatic men reported more difficulty with urinary incontinence (Adjusted mean ratio (AMR): 0.96, 95% CI: 0.96-0.97), urinary irritation (AMR: 0.95, 95% CI: 0.95-0.96), bowel function (AMR: 0.97, 95% CI: 0.97-0.98), sexual function (AMR: 0.90, 95% CI: 0.88-0.92), and vitality/hormonal function (AMR: 0.96, 95% CI: 0.96-0.96) than PSA-detected men. Differences were consistent across respondents of differing age, stage, Gleason score and treatment type.

**Conclusion:**

Prostate cancer survivors presenting symptomatically report poorer functional outcomes than PSA-detected survivors. Differences were not explained by socio-demographic or clinical factors. Clinicians should be aware that men presenting with symptoms are more likely to report functional difficulties after prostate cancer treatment and may need additional aftercare if these difficulties persist. Method of presentation should be considered as a covariate in patient-reported outcome studies of prostate cancer.

## Background

Prostate cancer is the most commonly diagnosed cancer among men from Western countries and the second most common cancer among men worldwide [1, 2]. The overwhelming majority of men diagnosed with prostate cancer either present symptomatically to a clinician or by a prostate specific antigen (PSA) test as part of a general/private health check [3]. Controversy exists regarding the PSA test as a screening test for prostate cancer, with conflicting conclusions regarding the test’s ability to lower prostate cancer related mortality [4–6]. In the absence of evidence of the benefits of PSA testing all men aged 50 and over in the UK may have a PSA test if they request it after being made aware of its potential implications [7, 8]. In England approximately 9 out of 100 men have a PSA test each year (2010-2011, aged 45-84), with only one quarter of these men having relevant urinary symptoms in the 12 months prior to the test [9]. Of the men having a PSA test for any reason 12% were referred to secondary care within 14 days and 4% had a diagnosis of prostate cancer [9]. Potential implications of PSA-testing for asymptomatic prostate cancer include the problem that 23-43% of these cancers are clinically insignificant, while men with clinically significant disease may be aware of their diagnosis for longer but with no survival benefit [10, 11]. Regardless of these issues all men diagnosed with both PSA-detected and symptomatic prostate cancer have to decide upon monitoring or a course of treatment, the latter of which can be associated with side effects [12–16].

Previous research found asymptomatic men diagnosed by PSA testing were younger, more affluent, had fewer comorbidities, earlier stage disease, lower Gleason score, and were more likely to have radical prostatectomy or brachytherapy/radiotherapy compared with symptomatically diagnosed men [17–19]. Although the characteristics of PSA-detected men are well documented, investigations of differences in outcomes between PSA-detected and symptomatic men are limited. Previous studies found PSA-detected men have better progression free survival after radical prostatecomy [19] lower disease specific mortality [20], reduced risk of metastases [20] and report better psychological wellbeing [17] compared to symptomatically diagnosed men after adjustment for stage and treatment. Although urinary incontinence, impotence, bowel problems and fatigue were also found to be more common among symptomatic men, these differences were unadjusted for treatment or stage [17].

In this study differences in prostate cancer related functional outcomes between symptomatic and PSA-detected survivors are investigated as part of the Life After Prostate Cancer Diagnosis (LAPCD) study [21], a population based study of over 35,000 men diagnosed with prostate cancer 18-42 months previously, the results of which have been previously reported for specific stage and treatment types [12]. In addition, for the first time, we investigate whether differences in functional outcomes between symptomatic and PSA-detected survivors can be explained by socio-demographic or clinical characteristics, and thus provide an assessment of the degree to which method of presentation is associated with quality of life after prostate cancer treatment.

## Methods

### Subjects/patients

Fifty-eight thousand nine hundred thirty men living with a prostate cancer diagnosis in the previous 18-42 months were surveyed by postal questionnaire throughout the United Kingdom (UK) between October 2015 and November 2016. In England, Wales and Northern Ireland (NI) national, population-based cancer registries were used to identify eligible men, while in Scotland cancer registry verified hospital activity data was used. The time period of 18-42 months was chosen as it reflects the point when initial treatment is complete and side effects have begun to stabilise [16].

### Survey

The survey asked men a range of socio-demographic questions including marital status, employment status, comorbidities and height and weight which were used to calculate body mass index (see Additional file 1: Table S1 for categories used). Men were asked to indicate which of the following treatment type(s) they received/were receiving: surgery, external-beam radiotherapy (EBRT), androgen deprivation therapy (ADT), brachytherapy, systemic (chemotherapy, abiraterone, enzalutamide), other treatment and monitoring only (active surveillance and watchful waiting). To determine method of presentation men were asked how they were diagnosed with prostate cancer and invited to tick all options that applied to them and/or provide text comments (Additional file 2). Using both the tick box responses and free-text comments men were assigned to one of four groups:

1. PSA-detected: Men ticked that they had no symptoms and either asked for or were offered a PSA test either by their GP or as part of a private health check;
2. Symptomatic: Men ticked that they attended their GP with urinary or other symptoms, or mentioned such symptoms in the free text box. These men may or may not also have had a PSA test;
3. Other: Men ticked that they presented via another method only;
4. Unknown: Men did not tick any box or did not provide text comments that allowed assignment to one of the previous three categories.

The 26-item Expanded Prostate cancer Index Composite (EPIC-26) [22] was used to assess health-related functional outcomes. Similar to previous studies [12, 23], reported prevalence experiencing specific problems was based upon the proportion of men reporting moderate/big problems (or equivalents such as poor/very poor) to individual questions. Based upon the EPIC-26 scoring instructions [24] the questions were divided up into five domains (urinary incontinence, urinary irritation/obstruction, bowel function, sexual function, and vitality/hormonal function), with summary scores for each domain calculated by averaging standardised scores assigned to responses to each question. All domains are scored out of a total of 100, with a lower score representing more problems/poorer functioning.

Age, nation of residence and deprivation quintile (based on area of residence at time of diagnosis) were extracted from national cancer registries. Stage and Gleason score at diagnosis were also provided by cancer registries as measures of disease severity. Stage was based upon the TNM classification, while Gleason score was categorised as 2-6 (slow growing cancer), 7 (intermediate risk of aggressive cancer) and 8-10 (cancer more likely to spread rapidly).

### Statistical analysis

As a result of variation in item completeness between PSA-detected and symptomatic men (Additional file 1: Table S1) all missing data items, except for method of presentation, were imputed in order to reduce any bias that may result from only including cases with complete data [25, 26]. Multiple imputation with chained equations [27, 28] was utilised with all socio-demographic, clinical characteristics, and EPIC-26 outcomes included. A secondary analysis including complete cases only (i.e. those men for whom all data items were complete) was also conducted.

The characteristics of symptomatic and PSA-detected men were compared using multivariable binary logistic regression with age at diagnosis, nation, deprivation, ethnicity, employment status, marital status, number of co-morbidities, body mass index, stage at diagnosis, Gleason score at diagnosis, and treatment type included in the model.

Mean functional outcome scores for symptomatic and PSA-detected men were initially compared using two way ANOVA, with method of presentation, a second characteristic (either age, stage, Gleason score or treatment type) and an interaction term between the two included. Multivariable log-linear regression with robust standard errors was utilised to adjust for different case mix between the two groups, with mean score ratios reported. Age at diagnosis, nation, deprivation quintile of residence, ethnicity, employment status, marital status, number of co-morbidities, body mass index, stage at diagnosis, Gleason score at diagnosis and treatment type were included in the models for each outcome. Given clinical interest in patients with particular clinical characteristics and possible interaction identified by the two-way ANOVA between method of presentation and these characteristics, further sub group analysis was conducted. Respondents were stratified by age, stage, Gleason score and treatment types, with the multivariable analysis also run for each strata. The Bonferonni correction was applied in the assessment of statistical significance given that comparisons were made across multiple outcomes. All analysis was conducted using Stata v14.

## Results

A total of 35,823 men responded to the survey, a response rate of 60.8%. Of these 11,210 (31.3%) were PSA-detected, 21,378 (59.7%) were symptomatic and 9.0% presented via an alternative method (e.g. referral from urologist, emergency admission to hospital) or with an unknown method of presentation. Age, stage, Gleason score and treatment type by presentation method are presented in Table 1, with additional socio-demographic characteristics presented in Additional file 1: Table S2. The distribution of all socio-demographic and clinical characteristics included in these tables varied significantly (all *p* < 0.001) by method of presentation. The 3235 men who presented with an alternative/unknown method of presentation were subsequently excluded leaving 32,588 PSA-detected/symptomatic prostate cancer survivors available for analysis.

**Table 1:** Respondent characteristics by method of presentation

Respondent characteristics	All respondents (*n* = 35,823)	Method of presentation			
		PSA-detected (*n* = 11,210)	Symptomatic (*n* = 21,378)	Other (*n* = 1821)	Unknown (*n* = 1414)
Age at diagnosis					
< 54	3.9%	4.0%	3.7%	5.7%	3.5%
55-64	23.8%	24.4%	23.7%	26.1%	17.5%
65-74	47.4%	48.9%	47.0%	46.1%	44.7%
75+	24.9%	22.7%	25.6%	22.1%	34.4%
Stage					
I/II	64.0%	71.2%	60.1%	65.3%	64.4%
III	23.3%	22.0%	24.3%	20.7%	21.6%
IV	12.7%	6.7%	15.6%	14.0%	14.0%
Gleason score					
2-6	29.0%	31.1%	27.7%	29.5%	30.8%
7	47.3%	51.4%	45.3%	48.1%	45.0%
8-10	23.7%	17.5%	27.0%	22.3%	24.2%
Treatment type ^a^					
Any surgery	30.0%	31.9%	28.9%	31.8%	28.9%
Any EBRT	38.9%	37.5%	40.4%	35.6%	32.5%
Any brachytherapy	8.6%	10.7%	7.6%	7.7%	9.2%
Any ADT	43.0%	37.0%	46.6%	42.3%	37.6%
Any systemic	5.0%	2.5%	6.2%	5.7%	4.9%
Any other	14.0%	15.0%	13.3%	12.4%	18.5%
Monitoring only	16.8%	18.1%	15.9%	17.3%	20.2%

Notes

*EBRT* External Beam Radiotherapy, *ADT* Androgen Deprivation Therapy, Systemic - Chemotherapy/Abiraterone/Enzalutamide

Additional respondent characteristics are available in Additional file 1: Table S2

^a^ Men may have more than one type of treatment

### Method of presentation

Prostate cancer survivors aged 65-74 were more likely than those aged under 55 to be PSA-detected (Adjusted odds ratio (AOR): 0.83, 95% confidence interval (CI): 0.72-0.95), while compared to men diagnosed at stage I/II, men diagnosed at stage III (AOR: 1.20, 95% CI: 1.12-1.28) or stage IV (AOR: 2.03, 95% CI: 1.82-2.27) were more likely to have been symptomatic. Compared to those with Gleason score 2-6, men with a Gleason score of 8-10 were more likely to present symptomatically (AOR: 1.12, 95% CI: 1.03-1.22), while men with a score of 7 were less likely to have presented symptomatically (AOR: 0.86, 95% CI: 0.80-0.92). Men receiving brachytherapy (AOR: 0.77, 95% CI: 0.70-0.85) were more likely to have been PSA-detected, while men receiving ADT (AOR: 1.22, 95% CI: 1.14-1.30) or systemic treatment (AOR: 1.64, 95% CI: 1.43-1.89) were more likely to have been symptomatic (Table 2).

**Table 2:** Age, stage, Gleason score and treatment received for PSA-detected compared to symptomatic prostate cancer survivors^#^

Respondent characteristics	Proportion PSA-detected ^a^ (*n* = 11,210)	Proportion symptomatic ^a^ (*n* = 21,378)	Adjusted odds ratio (95% CI) of being symptomatic compared to PSA-detected ^b^ (*n* = 32,588)
All respondents (*n* = 32,588)	34.4%	65.6%	–
Age at diagnosis			
< 54	36.3%	63.7%	1.00
55-64	35.0%	65.0%	0.92 (0.81-1.05)
65-74	35.3%	64.7%	0.83 (0.72-0.95)*
75+	31.8%	68.2%	0.87 (0.75-1.01)
Stage			
I/II	38.3%	61.7%	1.00
III	32.2%	67.8%	1.20 (1.12-1.28)**
IV	18.5%	81.5%	2.03 (1.82-2.27)**
Gleason score			
2-6	37.0%	63.0%	1.00
7	37.3%	62.7%	0.86 (0.80-0.92)**
8-10	25.4%	74.6%	1.12 (1.03-1.22)*
Treatment type ^c^			
Any surgery	36.7%	63.3%	1.05 (0.98-1.13)
Any EBRT	32.7%	67.3%	1.04 (0.98-1.11)
Any brachytherapy	42.4%	57.6%	0.77 (0.70-0.85)**
Any ADT	29.4%	70.6%	1.22 (1.14-1.30)**
Any systemic	17.7%	82.3%	1.64 (1.43-1.89)**
Any other	37.1%	62.9%	1.00 (0.92-1.09)
Monitoring only	37.4%	62.6%	1.05 (0.96-1.16)

Notes

*EBRT* External Beam Radiotherapy, *ADT* Androgen Deprivation Therapy, Systemic - Chemotherapy/Abiraterone/Enzalutamide # Alive 18-42 months after diagnosis, CI: Confidence interval

* *p* < 0.05, ** *p* < 0.001

^a^ Denominator excludes other and missing method of presentation

^b^ Determined using multivariable logistic regression adjusted for other variables in the table plus nation, deprivation, number of comorbidities, BMI, ethnicity, marital status and employment status. An odds ratio greater than 1 represents a greater odds than the baseline (i.e. first) group of being symptomatic compared to PSA-detected

^c^ Men may have more than one type of treatment. Odds ratios for treatment type have a reference category of not receiving that treatment type (i.e. No surgery, No EBRT etc.)

### Unadjusted post-treatment outcomes (EPIC-26)

**Fig. 1:**
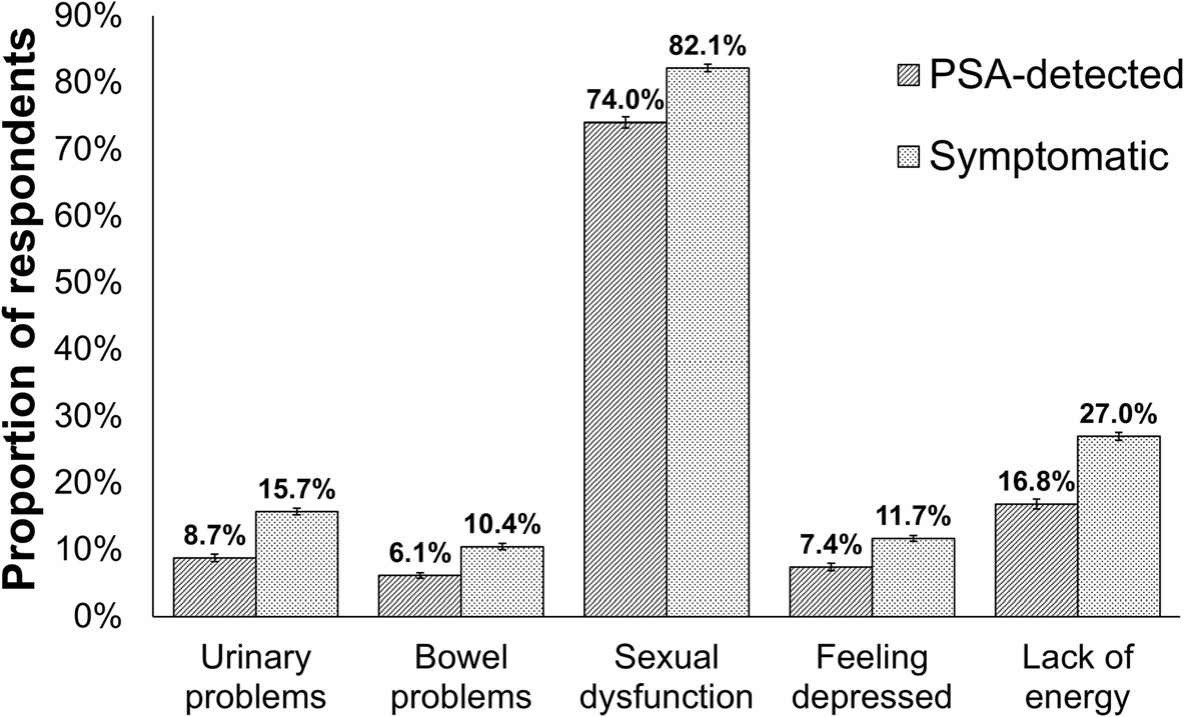
Proportion of PSA-detected and symptomatic prostate cancer survivors^#^ reporting moderate/severe* urinary, bowel and sexual problems, feeling depressed and lack of energy, measured using unadjusted individual items from the EPIC-26 questionnaire. Notes: See Additional file 1: Table S3 for further responses to individual questions, including confidence intervals and the results of statistical tests comparing patient groups. Error bars represent the 95% confidence intervals around each proportion. # Alive 18-42 months after diagnosis; * or equivalents such as poor/very poor

The proportion of PSA-detected men reporting moderate/big problems for each EPIC-26 question was significantly lower than for symptomatic men (*p* < 0.001), with the exception of problems with bloody stools, possibly due to the low reported frequency of this outcome (Fig. 1, Additional file 1: Table S3). Consequently mean outcome scores for each EPIC-26 domain were higher for PSA-detected men than symptomatic men (urinary incontinence: 84.0 vs 80.1; urinary irritation: 87.3 vs 82.5; bowel problems: 90.0 vs 86.6; sexual problems: 29.3 vs 23.1; vitality/hormonal problems: 83.6 vs 76.8; all *p* < 0.001), indicating that problems were reported less frequently (100 = no problems) by PSA-detected men. This relationship was present for all subgroups of patients defined by age, stage at diagnosis, Gleason score at diagnosis and treatment type (Additional file 1: Table S4).

### Case-mix adjusted post-treatment outcomes by method of presentation

After case-mix adjustment for clinical and socio-demographic characteristics, all functional outcome scores for symptomatic men were significantly lower than for PSA-detected men (all p < 0.001). The greatest relative difference was for sexual function, where the mean sexual function score was 10.0% lower (absolute difference in scores approximately 2.5 points) among symptomatic men (Adjusted mean ratio (AMR): 0.90, 95% CI: 0.88-0.92). The smallest relative difference was for bowel function which was on average 2.3% lower (absolute difference in scores approximately 2.0 points) among symptomatic men (AMR: 0.97, 95% CI: 0.97-0.98) (Table 3).

**Table 3:** Case mix adjusted ratio of mean functional outcome scores (EPIC-26) of PSA-detected compared to symptomatic prostate cancer survivors^c^

Respondent characteristics at diagnosis	Number of cases	Adjusted mean ratio (95% CI) – symptomatic vs. PSA-detected ^a^				
		Urinary incontinence	Urinary irritation/ obstruction	Bowel function	Sexual function	Vitality/ hormonal function
All respondents	32,588	0.96 (0.96-0.97)**	0.95 (0.95-0.96)**	0.97 (0.97-0.98)**	0.90 (0.88-0.92)**	0.96 (0.96-0.96)**
Age group						
Under 75	24,566	0.96 (0.95-0.96)**	0.95 (0.95-0.96)**	0.97 (0.97-0.98)**	0.91 (0.89-0.93)**	0.96 (0.95-0.96)**
75 and over	8017	0.97 (0.95-0.98)**	0.96 (0.95-0.97)**	0.98 (0.97-0.99)**	0.91 (0.86-0.96)*	0.96 (0.95-0.98)**
Stage						
I/II	17,818	0.96 (0.95-0.96)**	0.95 (0.95-0.96)**	0.97 (0.97-0.98)**	0.91 (0.88-0.93)**	0.96 (0.96-0.97)**
III	6643	0.97 (0.95-0.98)**	0.96 (0.95-0.97)**	0.98 (0.97-0.99)**	0.88 (0.83-0.94)**	0.96 (0.94-0.97)**
IV	3535	0.98 (0.96-1.00)	0.96 (0.95-0.98)**	0.99 (0.97-1.01)	0.86 (0.76-0.96)*	0.95 (0.92-0.97)**
Gleason score						
2-6	8083	0.97 (0.96-0.98)**	0.95 (0.94-0.96)**	0.97 (0.97-0.98)**	0.93 (0.90-0.96)**	0.97 (0.96-0.97)**
7	12,917	0.95 (0.95-0.96)**	0.96 (0.95-0.96)**	0.97 (0.96-0.98)**	0.88 (0.85-0.91)**	0.88 (0.85-0.91)**
8-10	6077	0.97 (0.96-0.99)*	0.96 (0.95-0.97)**	0.99 (0.98-1.00)	0.87 (0.80-0.93)*	0.95 (0.93-0.96)**
Treatment type ^b^						
Any surgery	9710	0.97 (0.96-0.99)*	0.96 (0.96-0.97)**	0.97 (0.97-0.98)**	0.93 (0.89-0.97)*	0.96 (0.96-0.97)**
Any EBRT	12,780	0.96 (0.95-0.97)**	0.96 (0.95-0.96)**	0.97 (0.97-0.98)**	0.84 (0.80-0.87)**	0.95 (0.94-0.96)**
Any ADT	14,016	0.96 (0.95-0.97)**	0.96 (0.95-0.96)**	0.98 (0.97-0.98)**	0.86 (0.82-0.90)**	0.95 (0.94-0.96)**
Monitoring only	5378	0.95 (0.94-0.96)**	0.94 (0.93-0.94)**	0.97 (0.97-0.98)**	0.91 (0.88-0.94)**	0.97 (0.96-0.98)**

Notes

*CI* Confidence Interval, *EBRT* External Beam Radiotherapy, *ADT* Androgen Deprivation Therapy

*p < 0.05, **p < 0.001 after Bonferroni correction for multiple comparisons

^a^ Determined using multivariable log-linear model adjusted for other variables in the table plus nation, deprivation, number of comorbidities, BMI, ethnicity, marital status and employment status. An adjusted mean score of less than one can be interpreted to mean that symptomatic patients have poorer functionality than PSA-detected patients

^b^ Men may have more than one type of treatment

^c^ Alive 18-42 months after diagnosis

This pattern was also present for those aged over and under 75 and for stage I/II, stage III and stage IV patients (all p < 0.001), with the exception of no significant difference in urinary incontinence or bowel function between symptomatic and PSA-detected stage IV patients. Outcomes for prostate cancer survivors with Gleason scores 2-6, 7 and 8-10 were also poorer among those who presented symptomatically with the exception of bowel function among those with Gleason score 8-10. Among men receiving surgery, EBRT, ADT or monitoring only, functional outcomes were also consistently poorer among symptomatic men (Table 3).

Similar results were found in the complete case analysis, with the exception of no significant difference in sexual function between symptomatic and PSA-detected men aged over 75, with stage III/IV disease or in receipt of surgery. This is likely due to the reduction in statistical power as the adjusted mean ratio is similar to that in the main analysis using imputed data (Additional file 1: Table S5).

## Discussion

This large UK wide population based study of over 35,800 prostate cancer survivors has enabled robust investigation of variations in functional outcomes by presentation method. We found that almost one third of survey responders reported presenting via a PSA test without experiencing any symptoms prior to diagnosis. Urinary, bowel, sexual and hormone-related problems are known to vary among prostate cancer patients depending upon treatment type [12–16]. To date, however, there has been limited investigation of whether these problems vary by method of presentation. We found that PSA-detected patients reported fewer urinary, bowel, sexual and hormone-related problems after treatment for their cancer and that these differences were independent of socio-demographic and clinical factors including treatment.

Uniquely with this large dataset we have also been able to investigate these patterns further by examining particular patient subgroups including age, stage, Gleason score and treatments received. Within each patient subgroup poorer outcomes were consistently reported by symptomatic men compared to those who were PSA-detected.

Differences between symptomatic and PSA-detected men are not limited to functional issues. Drummond et al. highlighted greater levels of depression, anxiety and stress among symptomatic men independent of treatment type and stage [17]. Similar to our study, Drummond et al. also reported higher levels of incontinence, impotence, bowel problems and fatigue among symptomatic men [17], although, unlike our study, these findings were not adjusted for treatment, stage and Gleason score at diagnosis.

Similar to other studies [17–19], PSA-detected men from this study had earlier stage of disease compared to symptomatically diagnosed men and were more likely to have brachytherapy than men who presented symptomatically, while symptomatic men were more likely to be treated with ADT. Given that adjusting for these factors did not eliminate differences in outcomes between different presentation methods, other factors are likely to be responsible for the better quality of life of PSA-detected men. A possible explanation is that PSA-detected men have better overall health and are less likely to have urinary, bowel and sexual problems prior to their prostate cancer diagnosis. PSA-detected men might also have been better supported already and have received (or been able to access) interventions to ameliorate functional problems. Further research is warranted to determine whether the differences reported here are due to better underlying physical and mental health of PSA-detected men or other systematic differences between the groups.

These findings suggest that men with symptomatic presentation need more follow up care for urinary, bowel and vitality/hormonal problems, regardless of whether these problems are due to treatment, background morbidity or lingering effects of presenting symptoms. Further research is required to assess whether investigation and treatment of symptoms prior to cancer patient management has a beneficial effect on functional outcomes. Importantly, these findings suggest method of presentation is a key factor in prostate cancer outcome studies and is an important covariate when comparing outcomes between patient groups with differing proportions of men who are symptomatic and PSA-detected.

### Strengths and limitations

Although this large population-based study had a good response rate and consisted of clinical data and patient reported outcomes, some limitations exist. Symptoms and treatments were self-reported and subjective rather than based upon clinical assessment or cancer-registration data. Also, while we have adjusted for clinical and socio-demographic factors, adjustments for treatment and background morbidity may be limited due to lack of information on treatment intensity (e.g. duration, frequency, radiation fraction, and ADT type), severity of co-morbidities or general health of patients before prostate cancer diagnosis. Additionally, the use of area based deprivation measures and employment status may not fully reflect each individual’s socio-economic status, thereby not fully capturing their health literacy and ability to negotiate health services.

It is also worth highlighting that while differences reported in this study are statistically significant this does not necessarily mean that they represent clinically significant or meaningful differences. Skolarus et al. [29] suggested clinically meaningful important differences (MID) for the EPIC-26 scores for comparisons at an individual level. In the event that these MIDs can be applied to populations, they suggest that only the differences in urinary irritation/obstruction and hormonal function for all patient subgroups, and differences for bowel function among younger men (aged under 64) may qualify as being clinically relevant. It is also important to note that conclusions about variations between patient groups may not necessarily reflect the experience of every individual patient.

## Conclusion

Prostate cancer survivors who present symptomatically have poorer urinary, bowel, sexual and vitality/hormonal function than those who were PSA-detected. Differences are not explained by the socio-demographic and clinical factors collected in the study, with this pattern observed for survivors of different age, diagnosed at early and late stage and in receipt of different treatment types. Health professionals should be aware that men presenting symptomatically report more functional difficulties after prostate cancer treatment, although this may be a result of poorer general health prior to diagnosis. Furthermore, method of presentation should be considered as a covariate in future prostate cancer outcome studies as quality of life varies by this characteristic which may thus partially explain differences in outcomes between patient groups that have different proportions of symptomatic prostate cancer.

## Supplementary information

**Additional file 1: Table S1.** Data item completeness by method of presentation. **Table S2.** Additional respondent characteristics by method of presentation. **Table S3.** Unadjusted responses to individual items from the EPIC-26 question set by method of presentation. **Table S4.** Mean functional outcome scores (EPIC-26) by respondent characteristics and method of presentation. **Table S5.** Case mix adjusted ratio of mean functional outcome scores (EPIC-26) of PSA-detected compared to symptomatic prostate cancer survivors – Complete case analysis.

**Additional file 2.** Life after prostate cancer diagnosis questionnaire.

## Data Availability

The datasets generated and/or analysed during the current study are not available publicly as eligible patients were informed at the time of the survey that their data would be stored securely and confidentially. The processes for accessing the data used are available from the corresponding author.

## Abbreviations

ADT: Androgen Deprivation Therapy
AMR: Adjusted Mean Ratio
ANOVA: Analysis of Variance
AOR: Adjusted Odds Ratio
CI: Confidence Interval
EBRT: External-beam Radiotherapy
EPIC: Expanded Prostate Cancer Index Composite
GP: General Practitioner
LAPCD: Life After Prostate Cancer Diagnosis
MID: Minimally Important Difference
NI: Northern Ireland
PSA: Prostate Specific Antigen
UK: United Kingdom
