# Supplementary material for: Quality of life among symptomatic compared to PSA-detected prostate cancer survivors - results from a UK wide patient-reported outcomes study
Source: BMC Cancer. 2019 Oct 15;19:947. doi: 10.1186/s12885-019-6164-5 (PMC6792209; doi:10.1186/s12885-019-6164-5)
Supplement: Supplementary file 1 — Additional file 1: Table S1. Data item completeness by method of presentation. Table S2. Additional respondent characteristics by method of presentation. Table S3. Unadjusted responses to individual items from the EPIC-26 question set by method of presentation. Table S4. Mean functional outcome scores (EPIC-26) by respondent characteristics and method of presentation. Table S5. Case mix adjusted ratio of mean functional outcome scores (EPIC-26) of PSA-detected compared to symptomatic prostate cancer survivors – Complete case analysis. [file 12885_2019_6164_MOESM1_ESM.docx]

## Quality of survival among symptomatic compared to PSA-detected prostate cancer survivors - Results from a UK wide patient-reported outcomes study: Supplementary data

### Table S1: Data item completeness by method of presentation

### Table S2: Additional respondent characteristics by method of presentation

### Table S3: Unadjusted responses to individual items from the EPIC-26 question set by method of presentation

### Table S4: Mean functional outcome scores (EPIC-26) by respondent characteristics and method of presentation

### Table S5: Case mix adjusted ratio of mean functional outcome scores (EPIC-26) of PSA-detected compared to symptomatic prostate cancer survivors – Complete case analysis

### Table S1: Data item completeness by method of presentation

| **Respondent characteristic** | **Proportion complete** | |
| --- | --- | --- |
|  | **PSA-detected** | **Symptomatic** |
| **Age** | 100.0% | 100.0% |
| **Deprivation indicator** | 97.7% | 97.6% |
| **Marital status** | 98.9% | 98.8% |
| **Employment status** | 96.5% | 96.9% |
| **Ethnicity** | 96.6% | 97.2% |
| **Body Mass Index** | 92.3% | 91.9% |
| **Stage at diagnosis** | 85.1% | 86.3% |
| **Treatment type** | 99.2% | 99.4% |
|  |  |  |
| **EPIC-26 domain scores** |  |  |
| **Urinary incontinence** | 90.2% | 88.4% |
| **Urinary irritation/obstruction** | 83.7% | 81.0% |
| **Bowel function** | 87.1% | 86.0% |
| **Sexual function** | 91.7% | 90.8% |
| **Vitality/Hormonal function** | 89.6% | 88.6% |

### Table S2: Additional respondent characteristics by method of presentation

|  | **All respondents**  **(n=35,823)** | **Method of presentation** | | | |
| --- | --- | --- | --- | --- | --- |
|  |  | **PSA test only**  **(n=11,210)** | **Symptomatic**  **(n=21,378)** | **Other**  **(n=1,821)** | **Unknown**  **(n=1,414)** |
| **Nation** |  |  |  |  |  |
| England | 85.0% | 86.0% | 84.4% | 84.6% | 87.8% |
| Wales | 7.0% | 7.4% | 6.9% | 7.9% | 5.4% |
| Scotland | 5.1% | 3.4% | 6.0% | 5.1% | 4.0% |
| Northern Ireland | 2.8% | 3.2% | 2.7% | 2.5% | 2.7% |
|  |  |  |  |  |  |
| **Deprivation** |  |  |  |  |  |
| Least deprived | 26.9% | 28.9% | 26.0% | 27.9% | 22.9% |
| Quintile 2 | 26.5% | 27.6% | 26.2% | 26.7% | 23.1% |
| Quintile 3 | 21.1% | 20.7% | 21.3% | 21.0% | 21.2% |
| Quintile 4 | 15.1% | 13.9% | 15.6% | 14.4% | 17.9% |
| Most deprived | 10.4% | 8.9% | 10.9% | 10.2% | 14.9% |
|  |  |  |  |  |  |
| **Ethnicity** |  |  |  |  |  |
| White | 97.0% | 96.6% | 97.2% | 98.1% | 95.7% |
| Non-white | 3.0% | 3.4% | 2.8% | 1.9% | 4.3% |
|  |  |  |  |  |  |
| **Employment status** |  |  |  |  |  |
| Employed | 19.8% | 22.5% | 18.4% | 22.1% | 15.8% |
| Retired | 77.3% | 75.4% | 78.2% | 75.0% | 81.3% |
| Unemployed | 2.2% | 1.4% | 2.6% | 1.9% | 2.1% |
| Other | 0.8% | 0.7% | 0.8% | 1.0% | 0.7% |
|  |  |  |  |  |  |
| **Marital status** |  |  |  |  |  |
| Married/Civil partnership | 80.3% | 81.7% | 79.8% | 80.1% | 75.7% |
| Divorced/Separated | 7.5% | 7.4% | 7.6% | 7.3% | 8.5% |
| Widowed | 8.2% | 7.3% | 8.6% | 7.7% | 11.4% |
| Single | 4.0% | 3.6% | 4.0% | 4.9% | 4.4% |
|  |  |  |  |  |  |
| **Number of co-morbidities** |  |  |  |  |  |
| None | 29.0% | 32.1% | 27.5% | 29.8% | 27.4% |
| 1 co-morbidity | 35.0% | 36.8% | 34.2% | 33.1% | 35.0% |
| 2 co-morbidities | 20.0% | 18.5% | 20.7% | 19.8% | 21.1% |
| 3 co-morbidities | 8.9% | 7.2% | 9.7% | 9.7% | 8.0% |
| 4 or more co-morbidities | 7.2% | 5.4% | 8.0% | 7.6% | 8.4% |
|  |  |  |  |  |  |
| **Body Mass Index (BMI)** |  |  |  |  |  |
| Under/healthy weight (<25) | 31.1% | 32.1% | 30.5% | 31.7% | 32.6% |
| Overweight (25-30) | 47.8% | 48.8% | 47.5% | 46.7% | 46.5% |
| Obese (30+) | 21.0% | 19.1% | 22.0% | 21.5% | 20.9% |

### Table S3: Unadjusted responses to individual items from the EPIC-26 question set by method of presentation

| **EPIC-26 item** | **Proportion reporting problems on each EPIC-26 question (95% CI)** | | |
| --- | --- | --- | --- |
|  | **PSA-detected (n=11,210)** | **Symptomatic (n=21,378)** | **p-value ^a^** |
| Leaked urine daily (or more) | 11.0% (10.4% - 11.6%) | 14.3% (13.8% - 14.8%) | <0.001 |
| Frequent dribbling/no urinary control | 5.7% (5.3% - 6.2%) | 9.0% (8.6% - 9.4%) | <0.001 |
| Pads used for urinary leakage | 11.7% (11.1% - 12.3%) | 13.8% (13.3% - 14.3%) | <0.001 |
| Moderate/big problems with dripping or leaking urine | 6.0% (5.6% - 6.5%) | 9.5% (9.1% - 9.9%) | <0.001 |
| Moderate/big problems with pain or burning on urination | 2.1% (1.7% - 2.4%) | 3.8% (3.5% - 4.0%) | <0.001 |
| Moderate/big problems with bleeding with urination | 0.6% (0.4% - 0.7%) | 1.2% (1.1% - 1.4%) | <0.001 |
| Moderate/big problems with weak urine stream or incomplete emptying | 8.5% (7.9% - 9.0%) | 15.6% (15.1% - 16.1%) | <0.001 |
| Moderate/big problems with need to urinate frequently during the day | 14.2% (13.5% - 14.8%) | 22.9% (22.3% - 23.5%) | <0.001 |
| Moderate/big problems with urinary function | 8.7% (8.2% - 9.3%) | 15.7% (15.2% - 16.2%) | <0.001 |
| Moderate/big problems with urgency to have a bowel movement | 7.8% (7.3% - 8.3%) | 11.4% (11.0% - 11.9%) | <0.001 |
| Moderate/big problems with increased frequency of bowel movements | 6.3% (5.8% - 6.7%) | 8.6% (8.2% - 9.0%) | <0.001 |
| Moderate/big problems with losing control of bowel movements | 3.7% (3.3% - 4.1%) | 5.2% (4.9% - 5.6%) | <0.001 |
| Moderate/big problems with bloody stools | 2.8% (2.5% - 3.2%) | 3.2% (3.0% - 3.5%) | 0.063 |
| Moderate/big problems with abdominal, pelvic, rectal or back passage pain | 2.8% (2.5% - 3.1%) | 6.1% (5.7% - 6.4%) | <0.001 |
| Moderate/big problems with bowel habits | 6.1% (5.7% - 6.5%) | 10.4% (10.0% - 10.9%) | <0.001 |
| Poor ability to have an erection | 74.6% (73.8% - 75.4%) | 82.3% (81.8% - 82.9%) | <0.001 |
| Poor ability to reach orgasm | 67.6% (66.7% - 68.5%) | 78.0% (77.4% - 78.6%) | <0.001 |
| Erections not firm | 83.8% (83.1% - 84.5%) | 89.1% (88.6% - 89.5%) | <0.001 |
| Erections unreliable | 80.3% (79.6% - 81.1%) | 86.5% (86.0% - 87.0%) | <0.001 |
| Poor ability to function sexually | 74.0% (73.2% - 74.8%) | 82.1% (81.6% - 82.7%) | <0.001 |
| Moderate/big problems with sexual function | 42.3% (41.3% - 43.2%) | 46.1% (45.4% - 46.8%) | <0.001 |
| Moderate/big problems with hot flushes | 12.8% (12.2% - 13.5%) | 20.3% (19.7% - 20.9%) | <0.001 |
| Moderate/big problems with breast tenderness/ enlargement | 4.3% (3.9% - 4.7%) | 7.0% (6.6% - 7.3%) | <0.001 |
| Moderate/big problems with feeling depressed | 7.4% (6.8% - 7.9%) | 11.7% (11.2% - 12.1%) | <0.001 |
| Moderate/big problems with lack of energy | 16.8% (16.1% - 17.6%) | 27.0% (26.4% - 27.6%) | <0.001 |
| Moderate/big problems with change in body weight | 11.3% (10.7% - 11.9%) | 17.1% (16.6% - 17.6%) | <0.001 |

Notes:

1. The p-value refers to the results of a z-test comparing the proportion reporting problems by method of presentation (symptomatic vs PSA-detected).

CI: Confidence interval

### Table S4: Mean functional outcome scores (EPIC-26) by respondent characteristics and method of presentation

| **Respondent characteristics** | **Mean functional outcome score (EPIC-26) (95% CI) ^a,b^ (n=32,588)** | | | | | | | | | |
| --- | --- | --- | --- | --- | --- | --- | --- | --- | --- | --- |
|  | **Urinary incontinence** | | **Urinary irritation/ obstruction** | | **Bowel function** | | **Sexual function** | | **Vitality/hormonal function** | |
|  | **PSA-detected** | **Symptomatic** | **PSA-detected** | **Symptomatic** | **PSA-detected** | **Symptomatic** | **PSA-detected** | **Symptomatic** | **PSA-detected** | **Symptomatic** |
| **All respondents** |  | p<0.001 |  | p<0.001 |  | p<0.001 |  | p<0.001 |  | p<0.001 |
|  | 84.0  (83.6-84.3) | 80.1  (79.8-80.5) | 87.3  (87.0-87.6) | 82.5  (82.3-82.7) | 90.0  (89.7-90.3) | 86.6  (86.3-86.8) | 29.3  (28.8-29.9) | 23.1  (22.7-23.4) | 83.6  (83.2-83.9) | 76.8  (76.5-77.1) |
|  |  |  |  |  |  |  |  |  |  |  |
| **Age at diagnosis** |  | p<0.001 ^I^ |  | p<0.001 ^I^ |  | p<0.001 ^I^ |  | p<0.001 ^I^ |  | p<0.001 ^I^ |
| <54 | 82.5  (80.4-84.6) | 77.8  (76.0-79.5) | 89.0  (87.7-90.4) | 81.7  (80.4-83.0) | 91.2  (89.8-92.6) | 86.5  (85.2-87.9) | 49.3  (46.2-52.4) | 41.3  (38.9-43.7) | 84.4  (82.6-86.3) | 75.9  (74.2-77.6) |
| 55-64 | 83.7  (82.9-84.5) | 78.7  (78.0-79.3) | 88.7  (88.1-89.2) | 82.3  (81.8-82.8) | 92.1  (91.5-92.6) | 87.0  (86.4-87.5) | 38.4  (37.2-39.6) | 29.4  (28.6-30.2) | 85.8  (85.1-86.5) | 76.2  (75.5-76.8) |
| 65-74 | 83.9  (83.4-84.5) | 80.8  (80.4-81.3) | 87.3  (86.9-87.7) | 83.2  (82.8-83.5) | 89.8  (89.3-90.2) | 86.9  (86.6-87.3) | 28.0  (27.2-28.7) | 22.2  (21.8-22.7) | 84.1  (83.6-84.6) | 77.9  (77.5-78.3) |
| 75+ | 84.5  (83.8-85.3) | 80.6  (80.0-81.2) | 85.6  (85.0-86.2) | 81.5  (81.0-81.9) | 88.0  (87.3-88.6) | 85.5  (85.0-86.0) | 19.1  (18.3-19.9) | 16.1  (15.7-16.6) | 80.0  (79.2-80.7) | 75.3  (74.7-75.9) |
|  |  |  |  |  |  |  |  |  |  |  |
| **Stage** |  | p<0.001 ^I^ |  | p<0.001 |  | p<0.001 |  | p<0.001 ^I^ |  | p<0.001 ^I^ |
| I/II | 84.7  (84.2-85.1) | 80.0  (79.6-80.4) | 87.4  (87.1-87.7) | 82.5  (82.2-82.8) | 90.6  (90.3-91.0) | 87.2  (86.9-87.5) | 33.6  (32.9-34.2) | 28.5  (28.0-29.0) | 86.0  (85.6-86.4) | 81.0  (80.6-81.4) |
| III | 82.0  (81.1-82.9) | 79.6  (78.9-80.2) | 87.3  (86.7-88.0) | 82.8  (82.3-83.3) | 88.5  (87.7-89.2) | 85.3  (84.7-85.9) | 19.9  (19.0-20.8) | 16.3  (15.7-16.8) | 79.1  (78.2-80.0) | 72.7  (72.0-73.4) |
| IV | 82.8  (81.1-84.4) | 81.4  (80.6-82.2) | 86.0  (84.8-87.2) | 82.0  (81.4-82.6) | 87.9  (86.6-89.2) | 86.3  (85.6-87.0) | 15.6  (14.0-17.3) | 12.8  (12.3-13.4) | 72.8  (71.0-74.5) | 66.7  (65.9-67.5) |
|  |  |  |  |  |  |  |  |  |  |  |
| **Gleason score** |  | p<0.001 ^I^ |  | p<0.001 |  | p<0.001 ^I^ |  | p<0.001 ^I^ |  | p<0.001 ^I^ |
| 2-6 | 85.4  (84.7-86.1) | 81.0  (80.3-81.6) | 86.9  (86.4-87.4) | 81.9  (81.5-82.4) | 92.2  (91.7-92.7) | 89.0  (88.5-89.4) | 40.2  (39.0-41.3) | 34.9  (34.0-35.7) | 88.6  (88.1-89.1) | 84.1  (83.5-84.6) |
| 7 | 83.4  (82.8-83.9) | 79.2  (78.7-79.7) | 88.0  (87.6-88.4) | 83.2  (82.8-83.6) | 89.8  (89.4-90.2) | 86.1  (85.7-86.5) | 27.4  (26.7-28.1) | 21.9  (21.4-22.4) | 83.9  (83.4-84.4) | 77.8  (77.4-78.3) |
| 8-10 | 83.1  (82.1-84.1) | 80.9  (80.3-81.5) | 86.1  (85.4-86.8) | 81.9  (81.4-82.3) | 86.5  (85.6-87.5) | 84.9  (84.3-85.4) | 15.9  (15.0-16.8) | 13.0  (12.6-13.4) | 73.6  (72.6-74.6) | 67.5  (66.9-68.1) |

*Table S4 continued*

| **Respondent characteristics** | **Mean functional outcome score (EPIC-26) (95% CI) ^a,b^ (n=32,588)** | | | | | | | | | |
| --- | --- | --- | --- | --- | --- | --- | --- | --- | --- | --- |
|  | **Urinary incontinence** | | **Urinary irritation/ obstruction** | | **Bowel function** | | **Sexual function** | | **Vitality/hormonal function** | |
|  | **PSA-detected** | **Symptomatic** | **PSA-detected** | **Symptomatic** | **PSA-detected** | **Symptomatic** | **PSA-detected** | **Symptomatic** | **PSA-detected** | **Symptomatic** |
|  |  |  |  |  |  |  |  |  |  |  |
| **Surgery** |  | p<0.001 ^I^ |  | p<0.001 |  | p<0.001 |  | p<0.001 ^I^ |  | p<0.001 ^I^ |
| No | 88.0  (87.6-88.4) | 83.3  (83.0-83.7) | 86.1  (85.7-86.4) | 81.3  (81.0-81.6) | 88.7  (88.3-89.1) | 85.7  (85.4-86.0) | 30.9  (30.2-31.5) | 23.6  (23.2-24.0) | 81.5  (81.0-81.9) | 74.6  (74.2-74.9) |
| Yes | 75.4  (74.5-76.2) | 72.3  (71.6-73.0) | 90.0  (89.5-90.4) | 85.4  (84.9-85.8) | 92.7  (92.2-93.1) | 88.8  (88.4-89.2) | 26.1  (25.3-27.0) | 21.8  (21.2-22.4) | 88.0  (87.5-88.6) | 82.1  (81.6-82.6) |
|  |  |  |  |  |  |  |  |  |  |  |
| **EBRT** |  | p<0.001 |  | p<0.001 |  | p<0.001 |  | p<0.001 ^I^ |  | p<0.001 |
| No | 82.7  (82.2-83.2) | 79.0  (78.6-79.4) | 87.7  (87.4-88.1) | 82.6  (82.3-82.9) | 92.6  (92.3-92.9) | 89.2  (88.9-89.5) | 32.9  (32.2-33.6) | 26.3  (25.8-26.8) | 86.9  (86.5-87.3) | 80.1  (79.7-80.4) |
| Yes | 86.0  (85.5-86.6) | 81.8  (81.3-82.3) | 86.6  (86.2-87.1) | 82.3  (81.9-82.6) | 85.6  (85.1-86.2) | 82.7  (82.3-83.1) | 23.4  (22.6-24.1) | 18.3  (17.9-18.8) | 78.0  (77.4-78.6) | 71.9  (71.4-72.4) |
|  |  |  |  |  |  |  |  |  |  |  |
| **ADT** |  | p<0.001 |  | p<0.001 |  | p<0.001 ^I^ |  | p<0.001 ^I^ |  | p<0.001 |
| No | 82.6  (82.1-83.1) | 78.3  (77.9-78.8) | 88.0  (87.7-88.3) | 83.1  (82.7-83.4) | 91.9  (91.6-92.3) | 88.4  (88.1-88.8) | 34.7  (34.0-35.4) | 29.2  (28.7-29.7) | 88.8  (88.5-89.2) | 83.7  (83.3-84.0) |
| Yes | 86.3  (85.7-86.8) | 82.2  (81.8-82.7) | 86.1  (85.7-86.6) | 81.8  (81.5-82.2) | 86.6  (86.1-87.2) | 84.4  (84.0-84.8) | 20.2  (19.5-20.9) | 16.1  (15.7-16.5) | 74.7  (74.0-75.4) | 68.8  (68.4-69.3) |
|  |  |  |  |  |  |  |  |  |  |  |
| **Monitoring only** |  | p<0.001 ^I^ |  | p<0.001 ^I^ |  | p<0.001 |  | p<0.001 |  | p<0.001 ^I^ |
| No | 82.9  (82.5-83.4) | 79.5  (79.2-79.9) | 87.6  (87.3-87.9) | 83.0  (82.7-83.3) | 89.2  (88.9-89.5) | 85.9  (85.6-86.2) | 25.8  (25.2-26.3) | 19.9  (19.5-20.2) | 82.2  (81.8-82.6) | 75.1  (74.8-75.4) |
| Yes | 88.6  (87.8-89.3) | 83.3  (82.6-84.0) | 86.1  (85.5-86.8) | 79.8  (79.2-80.4) | 93.5  (93.0-94.0) | 90.1  (89.6-90.6) | 45.5  (44.0-46.9) | 40.1  (39.0-41.2) | 89.7  (89.1-90.3) | 85.5  (84.9-86.1) |

Notes:

1. A higher score indicates better functioning (100 = No problems).
2. The p-value refers to the significance of method of presentation (symptomatic vs PSA-detected) in a two-way ANOVA, with the respondent characteristic as second independent variable. “I” indicates a significant interaction (p<0.05) between method of presentation and the respondent characteristic.

EBRT - External Beam Radiotherapy, ADT - Androgen deprivation therapy, CI: Confidence interval.

### Table S5: Case mix adjusted ratio of mean functional outcome scores (EPIC-26) of PSA-detected compared to symptomatic prostate cancer survivors – Complete case analysis

| **Respondent characteristics** | **Urinary incontinence** | | **Urinary irritation/ obstruction** | | **Bowel function** | | **Sexual function** | | **Vitality\hormonal function** | |
| --- | --- | --- | --- | --- | --- | --- | --- | --- | --- | --- |
|  | **Number of cases** | **Adjusted mean ratio (95% CI) – symptomatic vs. PSA-detected ^a^** | **Number of cases** | **Adjusted mean ratio (95% CI) – symptomatic vs. PSA-detected ^a^** | **Number of cases** | **Adjusted mean ratio (95% CI) – symptomatic vs. PSA-detected ^a^** | **Number of cases** | **Adjusted mean ratio (95% CI) – symptomatic vs. PSA-detected ^a^** | **Number of cases** | **Adjusted mean ratio (95% CI) – symptomatic vs. PSA-detected ^a^** |
| **All** | 18,395 | 0.96 (0.95-0.97)** | 17,090 | 0.96 (0.95-0.96)** | 17,929 | 0.98 (0.97-0.98)** | 18,812 | 0.90 (0.88-0.93)** | 18,468 | 0.96 (0.96-0.97)** |
|  |  |  |  |  |  |  |  |  |  |  |
| **Age at diagnosis** |  |  |  |  |  |  |  |  |  |  |
| Under 75 | 14,890 | 0.96 (0.95-0.97)** | 13,982 | 0.96 (0.95-0.96)** | 14,619 | 0.98 (0.97-0.98)** | 15,311 | 0.91 (0.88-0.94)** | 14,995 | 0.96 (0.96-0.97)** |
| 75 and over | 3,505 | 0.96 (0.95-0.98)** | 3,108 | 0.96 (0.95-0.97)** | 3,310 | 0.98 (0.97-0.99)* | 3,501 | 0.93 (0.85-1.01) | 3,473 | 0.97 (0.95-0.98)** |
|  |  |  |  |  |  |  |  |  |  |  |
| **Stage** |  |  |  |  |  |  |  |  |  |  |
| I/II | 12,145 | 0.96 (0.95-0.97)** | 11,325 | 0.96 (0.95-0.96)** | 11,905 | 0.97 (0.97-0.98)** | 12,418 | 0.90 (0.88-0.93)** | 12,163 | 0.97 (0.96-0.97)** |
| III | 4,451 | 0.97 (0.95-0.98)** | 4,088 | 0.96 (0.95-0.97)** | 4,274 | 0.98 (0.97-0.99)* | 4,557 | 0.90 (0.84-0.98) | 4,449 | 0.95 (0.94-0.96)** |
| IV | 1,799 | 0.98 (0.95-1.01) | 1,677 | 0.97 (0.95-0.99)* | 1,750 | 1.01 (0.99-1.03) | 1,837 | 0.88 (0.75-1.03) | 1,856 | 0.96 (0.93-0.99) |
|  |  |  |  |  |  |  |  |  |  |  |
| **Gleason score** |  |  |  |  |  |  |  |  |  |  |
| 2-6 | 5,527 | 0.97 (0.96-0.98)** | 5,180 | 0.96 (0.95-0.97)** | 5,470 | 0.98 (0.97-0.98)** | 5,629 | 0.92 (0.89-0.96)** | 5,534 | 0.97 (0.96-0.98)** |
| 7 | 8,755 | 0.95 (0.94-0.96)** | 8,100 | 0.96 (0.95-0.97)** | 8,478 | 0.98 (0.97-0.98)** | 9,005 | 0.87 (0.84-0.91)** | 8,777 | 0.87 (0.84-0.91)** |
| 8-10 | 4,113 | 0.97 (0.95-0.99)* | 3,810 | 0.96 (0.94-0.97)** | 3,981 | 1.00 (0.98-1.01) | 4,178 | 0.87 (0.79-0.96)* | 4,157 | 0.95 (0.94-0.97)** |
|  |  |  |  |  |  |  |  |  |  |  |
| **Treatment type ^b^** |  |  |  |  |  |  |  |  |  |  |
| Any surgery | 6,643 | 0.97 (0.96-0.99)* | 6,124 | 0.97 (0.96-0.97)** | 6,479 | 0.98 (0.97-0.98)** | 6,821 | 0.93 (0.88-0.98) | 6,592 | 0.96 (0.96-0.97)** |
| Any EBRT | 8,926 | 0.96 (0.95-0.97)** | 8,290 | 0.96 (0.95-0.97)** | 8,674 | 0.98 (0.97-0.99)** | 9,154 | 0.82 (0.78-0.86)** | 9,029 | 0.95 (0.94-0.96)** |
| Any ADT | 9,535 | 0.96 (0.95-0.97)** | 8,906 | 0.96 (0.95-0.97)** | 9,366 | 0.98 (0.97-0.99)* | 9,714 | 0.85 (0.80-0.89)** | 9,798 | 0.95 (0.94-0.96)** |
| Monitoring only | 4,091 | 0.95 (0.94-0.96)** | 3,797 | 0.94 (0.93-0.95)** | 4,021 | 0.97 (0.96-0.98)** | 4,079 | 0.91 (0.87-0.94)** | 4,021 | 0.97 (0.96-0.98)** |

Notes:

1. Determined using multivariable log-linear model adjusted for other variables in the table plus nation, deprivation, number of comorbidities, BMI, ethnicity, marital status and employment status. An adjusted mean score of less than 1 can be interpreted to mean that symptomatic patients have poorer functionality than PSA-detected patients.
2. Men may have more than one type of treatment.

# Alive 18-42 months after diagnosis; *p<0.05, **p<0.001 after Bonferroni correction for multiple comparisons; CI - Confidence Interval; EBRT - External Beam Radiotherapy; ADT - Androgen Deprivation Therapy.
